# Nanoparticle-complexed antimiRs for inhibiting tumor growth and metastasis in prostate carcinoma and melanoma

**DOI:** 10.1186/s12951-020-00728-w

**Published:** 2020-11-23

**Authors:** Manfred Kunz, Madeleine Brandl, Animesh Bhattacharya, Lars Nobereit-Siegel, Alexander Ewe, Ulrike Weirauch, Doreen Hering, Anja Reinert, Hermann Kalwa, Juan Guzman, Katrin Weigelt, Sven Wach, Helge Taubert, Achim Aigner

**Affiliations:** 1 Department of Dermatology, Venereology and Allergology, Leipzig University Medical Center, Leipzig, Germany; 2 Rudolf-Boehm-Institute for Pharmacology and Toxicology, Clinical Pharmacology, University of Leipzig, Haertelstrasse 16–18, 04107 Leipzig, Germany; 3 Faculty of Veterinary Medicine, Institute of Anatomy, Histology and Embryology, Leipzig University, Leipzig, Germany; 4 Rudolf-Boehm-Institute for Pharmacology and Toxicology, University of Leipzig, Leipzig, Germany; 5 Department of Urology, Friedrich Alexander University Erlangen-Nürnberg, Erlangen, Germany; 6 Present Address: Department of Hematology, Oncology and Tumor Immunology, Charité-University Medical Center, Virchow Campus, Berlin, Germany

**Keywords:** Polyethylenimine, PEI, Antimir, PEI/antimiR nanoparticles, Therapeutic miRNA inhibition

## Abstract

**Background:**

MiRNAs act as negative regulators of gene expression through target mRNA degradation or inhibition of its translation. In cancer, several miRNAs are upregulated and play crucial roles in tumorigenesis, making the inhibition of these oncomiRs an interesting therapeutic approach. This can be achieved by directly complementary single-stranded anti-miRNA oligonucleotides (antimiRs). A major bottleneck in antimiR therapy, however, is their efficient delivery. The nanoparticle formation with polyethylenimine (PEI) may be particularly promising, based on the PEI’s ability to electrostatically interact with oligonucleotides. This leads to their protection and supports delivery. In the present study, we explore for the first time PEI for antimiR formulation and delivery. We use the branched low molecular weight PEI F25-LMW for the complexation of different antimiRs, and analyse tumor- and metastasis-inhibitory effects of PEI/antimiR complexes in different tumor models.

**Results:**

In prostate carcinoma, transfection of antimiRs against miR-375 and miR-141 leads to tumor cell inhibition in 2D- and 3D-models. More importantly, an in vivo tumor therapy study in prostate carcinoma xenografts reveals anti-tumor effects of the PEI/antimiR complexes. In advanced melanoma and metastasis, we identify by a microRNA screen miR-150 as a particularly relevant oncomiR candidate, and validate this result in vitro and in vivo. Again, the systemic application of PEI/antimiR complexes inhibiting this miRNA, or the previously described antimiR-638, leads to profound tumor growth inhibition. These effects are associated with the upregulation of direct miRNA target genes. In a melanoma metastasis mouse model, anti-metastatic effects of PEI/antimiR treatment are observed as well.

**Conclusions:**

We thus describe PEI-based complexes as efficient platform for antimiR therapy, as determined in two different tumor entities using in vivo models of tumor growth or metastasis. Our study also highlights the therapeutic relevance of miR-375, miR-141, miR-150 and miR-638 as target miRNAs for antimiR-mediated inhibition.
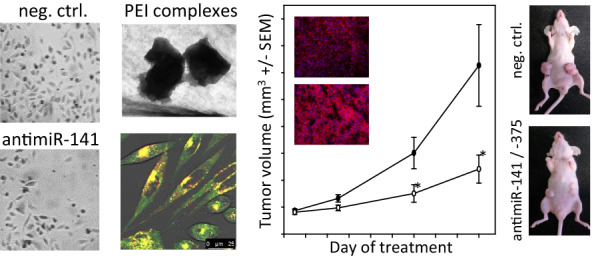

## Background

MiRNAs are small 21–23 nucleotide non-coding RNA molecules, which act as negative regulators of gene expression by binding to the 3′ untranslated region (3′ UTR) of mRNAs, leading to target mRNA degradation or inhibition of its translation. More than 2000 miRNAs exist in the human genome [1–3], with every miRNA being able to regulate a large number of genes, many of which are functionally related (https://microrna.sanger.ac.uk; https://pictar.bio.nyu.edu/).

Importantly, several miRNAs have been found to be upregulated in tumors and to exert oncogenic effects (see e.g. [4, 5]). They thus play a crucial role in the onset and progression of human cancer. The aberrant overexpression of these oncogenic miRNAs (oncomiRs) and their activity in malignancies, as demonstrated in a plethora of functional studies, underscore the relevance of miRNAs in tumorigenesis and tumor progression, and make them interesting therapeutic targets.

For their inhibition, the delivery of miRNA inhibitors including antimiRs, antagomirs, miRNA decoys or miRNA sponges has been employed. AntimiRs, i.e., anti-miRNA oligonucleotides for miRNA inhibition, are single-stranded oligonucleotides directly complementary to the miRNA to be inhibited [6]. As synthetic reverse complements, they prevent miRNA activity by competing with the target 3′UTR mRNA site for miRNA binding. The miRNA:antimiR hybridization leads to inactivation of the mature miRNA primarily through steric hindrance [7].

While expression vectors encoding antimiRs can be introduced by viruses for intracellular transcription of the miRNA inhibitor, viral approaches may raise several issues including potential safety problems with regard to insertional mutagenesis, induction of toxic immune responses or oversaturation of cellular miRNA/RISC machinery and difficulties in dosage. Thus, non-viral antimiR delivery approaches are preferred and also allow for using chemically modified antimiR oligonucleotides with enhanced nuclease stability, affinity and specificity. Beyond first generation anti-miRNA oligonucleotides employing 2′-O-Methyl RNA nucleotides (2′OMe) and phosphorothioate (PS) internucleotide linkages, this also includes locked nucleic acid (LNA) modifications [8]. LNA-modified oligonucleotides are known to be effective in functional miRNA inhibition with high affinity and specificity, accompanied by low toxicity and increased stability in vivo [9–11].

For their therapeutic exploration in vivo, however, the delivery of antimiR still represents a major bottleneck since enzymatic degradation, immunogenicity in the bloodstream as well as poor tissue penetration and cellular uptake of naked oligonucleotides render naked antimiRs rather inefficient. Since these issues can only partially be addressed by chemical modifications, nanoparticles may serve as delivery platforms, including liposomes or liposome-like stable nucleic acid lipid particles (SNALPs) [12], inorganic nanoparticles [13], exosomes [14] or polymeric nanoparticles. Among cationic polymers, polyethylenimine (PEI) can be considered as the gold standard for non-viral gene delivery due to its commercial availability and high transfection efficiency. It is able to electrostatically interact with oligonucleotides [15]. PEIs are linear or branched, synthetic polymers of variable molecular weights [16–18]. Upon nanoplex formation, RNAs have been shown to be completely protected against degradation and to be delivered and intracellularly released. Notably, this also applies to small RNA molecules like ribozymes, siRNAs or miRNAs [18–22]. While low molecular weight PEIs are preferable from the toxicological viewpoint, they may suffer from poor efficacy, especially with regard to small RNA molecules. This is particularly true for small single-stranded antimiRs. Previously, we introduced the 4–10 kDa branched PEI F25-LMW [18] and demonstrated its therapeutic potential for PEI-mediated siRNA or miRNA delivery in therapeutic in vivo applications [18–25].

For antimiR delivery, PEIs have been explored mainly as one component for modifying inorganic nanostructures like Au nanocages [26, 27], in co-polymers or in multifunctional nanocomplexes [28–31]. While PEG-shielded carboxymethyl PEI nanogels [32] or disulphide-crosslinked PEIs [33] have been described as well, in another study the branched 25 kDa PEI “gold standard” performed only poorly in terms of complex stability, efficacy and biocompatibility [34].

In the present study, we describe for the first time the use of PEI F25-LMW as an uncomplicated system for antimiR complexation and delivery. Using different antimiRs, we analyse its efficacy in different tumor models, with regard to tumor- and metastasis-inhibitory effects. This also demonstrates the broader applicability of PEI/antimiR complexes for miRNA inhibition.

## Results

### Tumor cell-inhibitory effects of miR-375/miR-141 inhibition

**Fig. 1:**
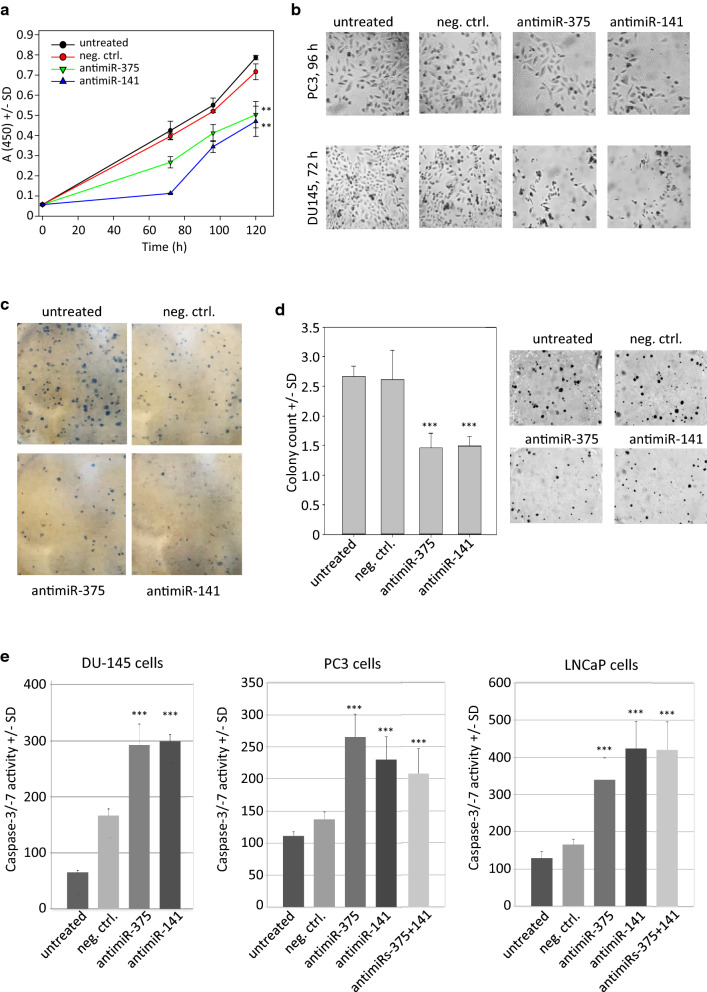
AntimiR-375 and antimiR-141 reduce tumor growth of prostate cancer in vitro and in vivo.** a** LNCaP prostate cancer cells were cultured under standard culture conditions and exposed to antimiR-375 or antimiR-141. Cell proliferation was measured using the colorimetric WST-1 assay. **b** PC3 and DU145 prostate cancer cells were transfected with antimiR-375 or antimiR-141 and grown at low density and stained with Hematoxylin. **c**,** d** DU145 prostate cancer cells were transfected with antimiRs and analyzed for anchorage-dependent (**c**, on plastic) and anchorage-independent (**d**, in soft agar) colony formation capacity. **e** LNCaP, PC3 and DU145 prostate cancer cells were transfected with antimiRs as above and caspase activity was measured as a marker for apoptosis induction

Based on previous studies on the potential roles of miR-375 and miR-141 as oncogenic miRNAs [35–38], we first assessed the effects of the antimiR-mediated miRNA inhibition in 2D and 3D cell growth in different in vitro models of prostate carcinoma. Transient transfection of LNCaP cells with antimiRs led to a substantial reduction in cell proliferation as compared to untreated or negative control transfected cells (Fig. 1a). Effects were more profound in the case of antimiR-141, with an almost complete inhibition of cell growth over the first 72 h post transfection. Similar inhibitory effects were observed in PC3 and in DU145 cells, with again antimiR-141 transfection leading to a more profound decrease in cell proliferation (Fig. 1b). This was paralleled by reduced DU145 colony formation on plastic (Fig. 1c). Switching to a 3D system closer to the in vivo situation, soft agar assays were performed. In this assay, single cells are seeded in a soft agar matrix, thus having the chance to grow anchorage-independently (i.e., not on a plastic surface as in classical 2D culture) and to form 3D colonies. This resembles much closer the in vivo conditions of cell growth. Upon plating pre-transfected DU-145 cells, a ~ 50% reduction of soft agar colony counts upon specific antimiR transfection was observed compared to negative controls (untreated or negative control transfected cells; Fig. 1d). Notably, in this assay inhibitory effects of antimiR-141 and antimiR-375 were comparable. Tumor cell-inhibitory effects may be due to increased apoptosis upon specific antimiR transfection. The extrinsic and the intrinsic apoptosis pathway rely on increased caspase-3/-7 activity, which was analyzed next. Indeed, a ~ twofold caspase-3/-7 activation was observed in all three cell lines and for both specific antimiRs (Fig. 1e).

### Formation and characterization of PEI/antimiR complexes

**Fig. 2:**
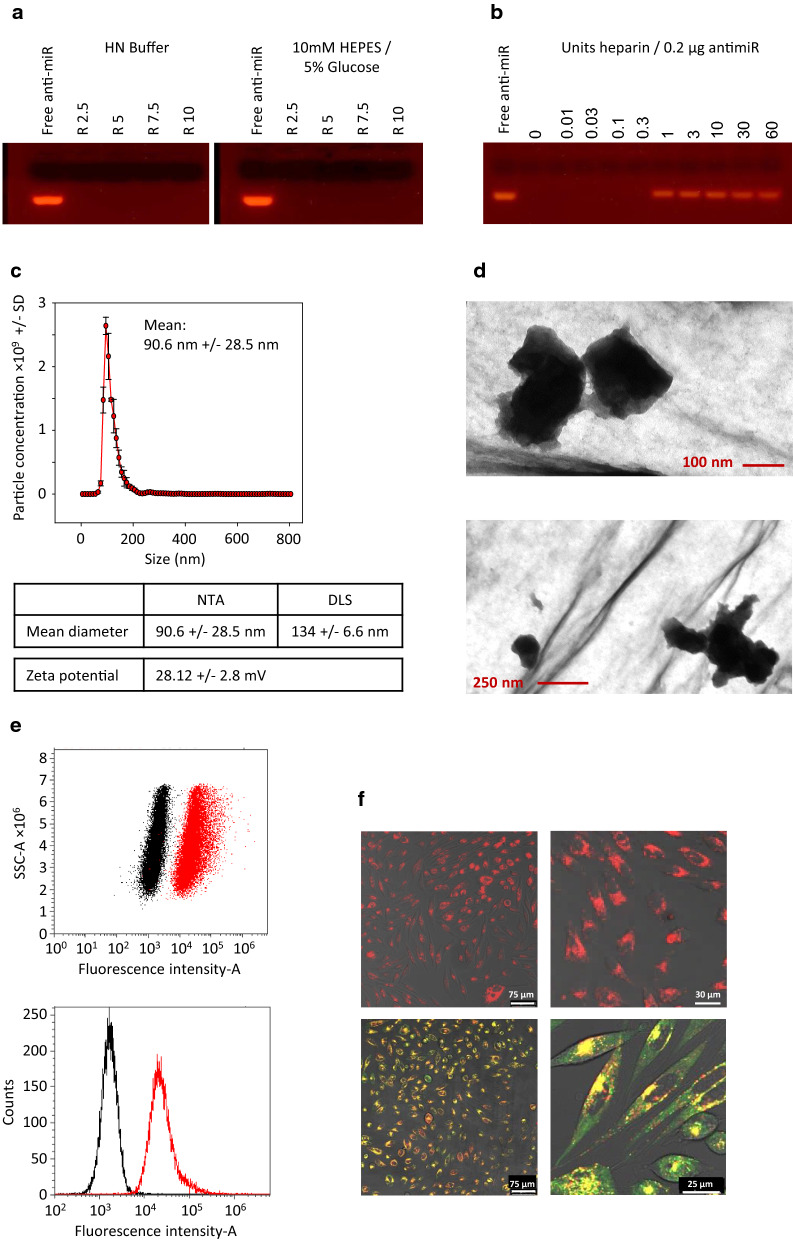
Nanoparticle complex stability and transfection rates. **a** For the determination of complexation efficacy by agarose gel electrophoresis, 0.5 µg antimiR were complexed with PEI at the different weight ratios. The complexes were mixed and separated on a 2% agarose gel pre-stained with Sybr™ Gold. **b** Complex stabilities were measured by heparin displacement assay. **c** PEI/antimiR complex sizes were determined by Dynamic Light Scattering (DLS). **d** Transmission electron microscopy (TEM) of PEI/antimiR complexes. **e,f** Uptake of PEI complexes containing Cy3-labeled antimiR was analyzed by flow cytometry and confocal microscopy

For the therapeutic in vivo application of small RNAs, formulations are necessary to avoid RNA degradation and rapid renal clearance as well as for mediating RNA delivery to the target tissue and cellular uptake. We have previously established polymeric, polyethylenimine (PEI)-based nanoparticles for siRNA or miRNA delivery, which was now extended towards antimiRs. Despite the fact that molecular weights of single-stranded 20-mer antimiRs (i.e., comprising only 20 nucleotides) are even smaller compared to double-stranded siRNAs, antimiRs were efficiently complexed already at low mass ratios. More specifically, a PEI: antimiR mass ratio of 2.5 was sufficient for full complexation, as indicated by the absence of a free antimiR band in gel electrophoresis (Fig. 2a). This was independent of the complexation conditions and thus also true for low salt buffer conditions (Fig. 2a, right), which generally lead to less efficient complexation and smaller complexes. Complexes were also found to be stable, as determined by heparin displacement assay. Complex decomposition required 1 unit heparin/0.2 µg antimiR for antimiR release, with no signs of partial complex destabilization at lower heparin concentrations (Fig. 2b). This result was identical to siRNA release from PEI/siRNA complexes (data not shown).

While non-modified siRNAs are prone to degradation, the chemical modification of the antimiR led to its stability against RNase. Free antimiR retained its integrity upon incubation with RNase or FCS, with less defined gel bands in the presence of FCS indicating its profound interaction with serum proteins (Additional file 2: Fig. S2a, lanes 1–4). In contrast, siRNA showed less interaction with serum proteins but partial or full degradation in the presence of FCS or RNase, respectively (Additional file 2: Fig. S2b). Both molecules were fully complexed by PEI F25-LMW and the complexes remained stable also in the presence of FCS (Additional file 2: Fig. S2a, b, right lanes). Upon heparin treatment, both complexes released their fully intact RNA cargo (Additional file 2: Fig. S2a, b, center lanes). AntimiRs and PEI/antimiR complexes remained also intact under conditions present in the lysosome. When incubating antimiRs with artificial lysosomal fluid (ALF), no degradation was observed (Additional file 2: Fig. S2c, left lanes). Also, PEI/antimiR complexes remained stable under these conditions, and only released the intact antimiR upon addition of heparin (Additional file 2: Fig. S2c, lanes 3–5). Taken together, this demonstrates the stability of complexes and antimiRs under relevant biological conditions.

By nanoparticle tracking analysis, PEI/antimiR complex sizes were determined at a rather distinct size of ~ 90 nm, with little variation (Fig. 2c, upper panel). This was largely confirmed by Dynamic Light Scattering (DLS), which revealed slightly larger complexes with even less size distribution (Fig. 2c, lower panel). Similar results were also obtained by transmission electron microsopy (TEM; Fig. 2d) which showed asymmetric complexes which, at higher concentrations, tended to aggregate (Fig. 2d, left vs. right). Complex sizes in the range of ~ 130 nm, an irregular shape of the complexes, as visualized by TEM, and a positive zeta potential of ~ 28 mV, as determined by Phase Analysis Light Scattering (PALS; Fig. 2c, right), were very similar to previously described PEI/siRNA complexes (see e.g., [39, 40]). Concomitantly, efficient uptake of PEI/antimiR complexes containing Cy3-labeled antimiRs was observed. The analysis of PC3 cells at 48 h after transfection revealed a percentage of cells taking up complex of ~ 100%, as determined by flow cytometry (Fig. 2e). This was also confirmed by confocal microscopy (Fig. 2f, upper panel). The direct comparison of uptake efficacies also revealed that antimiR uptake was dependent on its prior complexation (Additional file 1: Fig. S1a). More specifically, flow cytometry upon incubation of PC3 cells with free FAM-labeled antimiR showed results indistinguishable from untreated cells while profound uptake was observed in the case of the PEI/antimiR complexes. This was in line with biological antimiR efficacy in inhibiting cell proliferation (Additional file 2: Fig. S2b). PEI-complexed antimiR-375 or antimiR-141 led to a > 55% reduction of viable cells after 5 d as compared to their PEI-complexed negative control counterparts, while no effect was observed upon treatment of the cells with the same amount of uncomplexed antimiRs (Additional file 2: Fig. S2b).

When analyzing cell uptake in more detail by parallel staining with LysoTracker green, co-localization of both signals (yellow signals in Fig. 2f, lower panel) as well as color separation were observed. The analysis of the colocalization using the Fiji software and the colocalization plugin revealed a Pearson correlation coefficient from 4 images (20× magnification) of 0.78 ± 0.022. This indicates complexes in the lysosomes and their release from the lysosome into the cytoplasm, respectively.

Taken together, these results demonstrate the formation of stable complexes, capable of delivering intact antimiRs into cells. These PEI/antimiR complexes were subsequently employed in vivo.

### In vivo tumor therapy study in prostate carcinoma xenografts

**Fig. 3:**
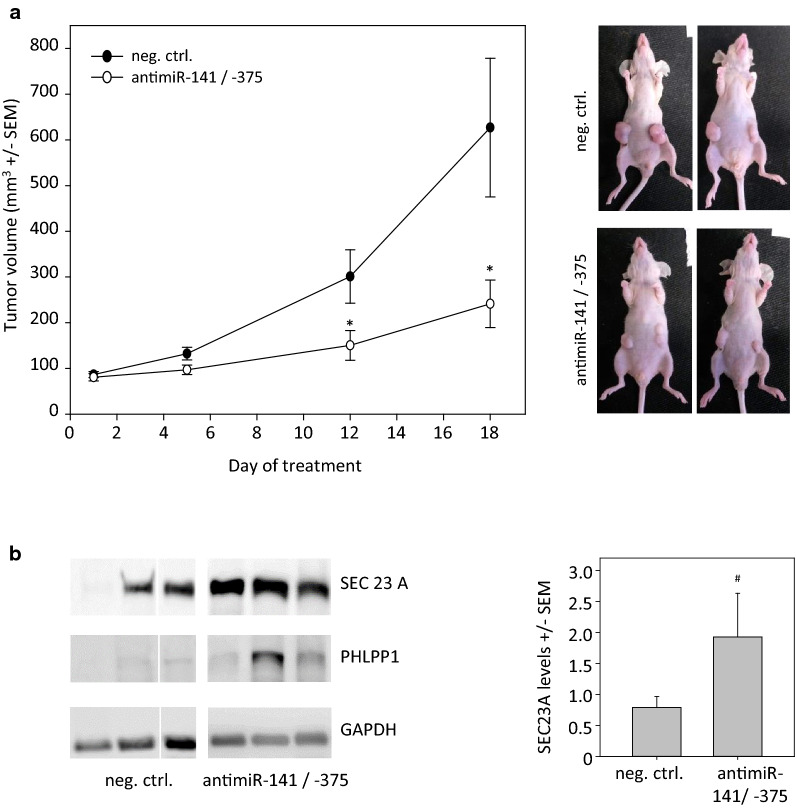
Treatment of mouse prostate cancer xenografts with PEI/antimiR complexes. **a** For the generation of tumor xenografts in athymic nude mice (Foxn1nu) 5 × 10^6^ PC3 cells in 150 µL PBS were injected subcutaneously into both flanks of mice. The PEI/antimiR complexes injected intraperitoneally every 2–3 days at the time points indicated in the figure. Right half of the picture shows representative pictures of mice injected with scrambled RNA (control) or antimiRs. **b** Analysis of tumor lysates by Western blotting, for alterations in the expression levels of miR-375 target genes SEC23A and PHLPP1. Left: representative samples; right: quantitation of all Western blot samples

Prostate carcinoma (PCa) xenografts were generated by subcutaneous (s.c.) injection of PC3 cells into athymic nude mice. Upon establishment of tumors > 80 mm^3^ with solid growth kinetics, mice were randomized into two groups and treated by intraperitoneal injection of 10 µg PEI-complexed antimiR-141 + antimiR-375 or PEI-complexed negative control RNA every 2–3 days at the time points indicated in the figure. In this experimental model, the intraperitoneal administration route was preferred over i.v. injection since we have shown previously that i.p. injected PEI-based complexes reach s.c. tumor xenografts more efficiently [24]. As shown in Fig. 3a, treatment with specific PEI/antimiR complexes led to a profound ~ 60% inhibition of tumor growth. No adverse effects of the repetitive injections were observed. Upon termination of the experiment, tumors were explanted and analyzed by Western blot for expression levels of miR-375 target genes SEC23A [36] and PHLPP1 [41]. While in both groups band intensities were found somewhat heterogeneous between individual samples, a profound upregulation of both proteins based on antimiR-mediated de-repression was observed (Fig. 3b).

### MicroRNA screen identifies miR-150 as an oncomiR candidate in melanoma

**Fig. 4:**
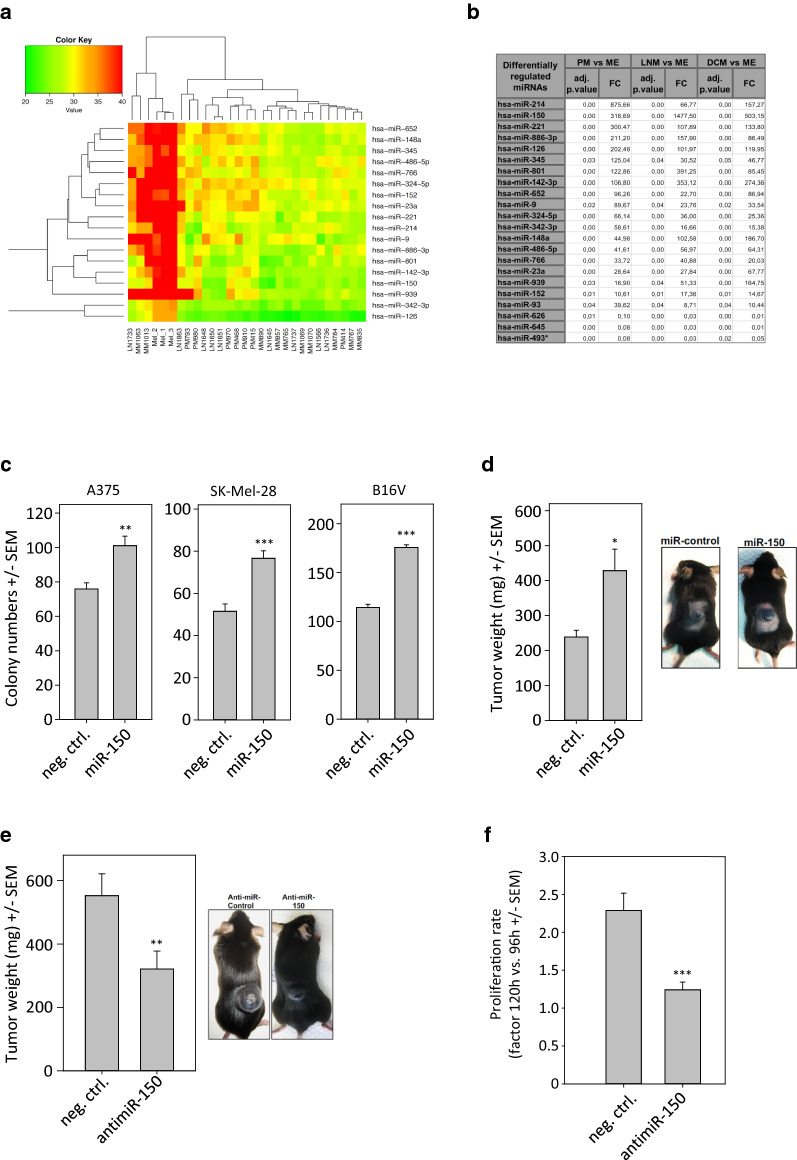
miR-150 drives melanoma growth in vivo. **a** TaqMan^®^ low-density arrays were used to measure the expression of 667 human miRNAs in a set of melanocytes, primary melanomas and cutaneous melanoma metastases. Relative expression of indicated miRNAs is shown. The heatmap shows high expression in green and low expression in red. **b** Fold changes (FC) of miRNA expression is indicated when comparing melanocytes (ME), primary melanomas (PM), lymph node metastases (LNM) and distant cutaneous metastases (DCM), respectively. **c** Melanoma cells were transfected with miR-150 and seeded at low density. Colony formation was counted by light microscopy. **d** miR-150 was overexpressed in B16V mouse melanoma cells after transfection of the microRNA mimic, and tumor cells were injected into flanks of C57BL6N mice. Tumors were surgically removed after 14 days and weighted. **e** B16V melanoma cells were transfected using antimiRs against miR-150 or antimiR-control oligos. Mice were sacrificed after 14 days and surgically removed tumors were weighted. **f** LOX cells were transfected with antimiRs and analyzed for growth rates (here: in the interval 96 h–120 h after transfection)

Based on our previous study on miRNAs in melanoma [42], we performed miRNA expression profiling of 670 different microRNAs in primary malignant melanomas (PM), lymph node metastases (LNM) and distant cutaneous metastases (DCM) using patient tissue samples, compared with that of freshly isolated primary melanocytes (ME; Fig. 4a). MiRNAs miR-214 and miR-150 showed the highest fold change in expression when comparing primary melanomas with melanocytes (ME), miR-150 showed the highest fold changes when comparing lymph-node metastases (LNM) and distant cutaneous metastases (DCM) with melanocytes (ME) (Fig. 4b). These data emphasized the potential role of miR-150 in melanoma progression. The role miR-214 in melanoma progression had been described in detail in earlier reports by another group [43]. Due to its high scores in primary melanoma as well as in metastasis, miR-150 was selected for further studies.

### miR-150 promotes melanoma growth in vitro and in vivo

Cells were transfected with a miR-150 expression vector, prior to seeding at low density and growth for 20 days. When overexpressing miR-150 in the three different melanoma cells lines SK-Mel-28, A375 and B16V in this way, a statistically significant increase in clonogenic cell growth was observed (Fig. 4c). More importantly, transient transfection of miR-150 in B16V cells also promoted melanoma growth in vivo (Fig. 4d). The miR-150-overexpressing versus negative control-transfected cells were s.c. transplanted at the back of syngeneic C57/BL6 mice and tumor xenografts were allowed to grow for 14 days. After this, the mice were sacrificed and volumes of the extracted tumors measured. While negative control (i.e., non-targeting control-RNA overexpressing) cells showed moderate tumor formation capacity when engrafted into mice, overexpression of miR-150 led to significantly increased tumor volumes (Fig. 4d).

More important, however, is the reverse experiment addressing the question if blocking of miR-150 might lead to melanoma cell inhibition and attenuated tumor growth. To address this issue, 5 × 10^6^ B16F10 melanoma cells were transfected with antimiR-150 or with a negative control RNA, prior to s.c. injection into the back of syngeneic C57/BL6 mice and tumor growth for 28 days. After this, the mice were sacrificed and volumes of the extracted tumors measured. Indeed, inhibition of miR-150 led to significantly reduced tumor growth compared to the control injected tumors (Fig. 4e). The tumor cell-inhibitory effect of antimiR treatment was also observed in vitro, when analyzing proliferation rates (Fig. 4f). This also indicates that the in vivo effects seen in Fig. 4e are not merely based on differences in tumor take, and identify miR-150 as a potential therapeutic target in melanoma. The LOX melanoma cells shown in Fig. 4f were also used for the subsequent in vivo therapy study.

### Therapeutic effects of PEI/antimiR complexes in melanoma xenografts in vivo

**Fig. 5:**
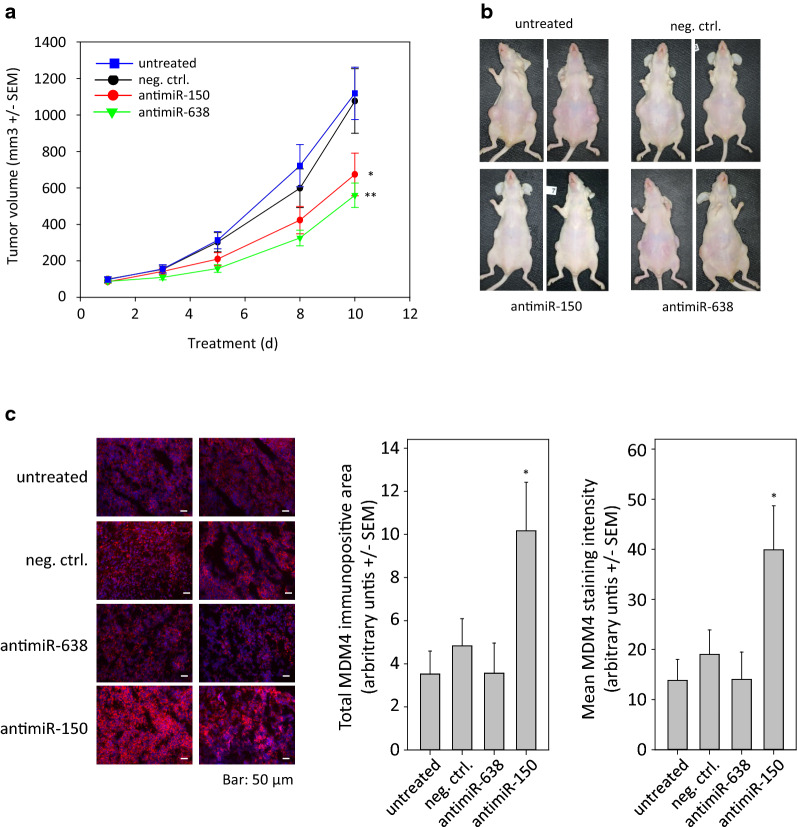
Treatment of local melanoma xenografts with antimiR/PEI complexes in mice. **a** For the generation of melanoma xenografts, 5 × 10^6^ LOX cells were injected subcutaneously into both flanks of athymic nude mice. The PEI/antimiR complexes were injected intraperitoneally every 2–3 days at the time points indicated in the figure. Tumor sizes were determined by measuring all three dimensions. **b** Representative pictures of mice. **c** Representative examples of immunofluorescence staining of melanoma xenografts with antibodies directed against the miR-150 target MDM4. The right panel shows the quantitation of immunofluorescence staining

Subcutaneous tumor xenografts from LOX cells were generated and, after establishment of tumors with solid growth kinetics, mice were randomized in negative control and treatment groups. The comparison between untreated and PEI-negative control RNA treated groups revealed the absence of non-specific effects of the treatment with PEI complexes (Fig. 5a). For these experiments, antimiRs targeting miR-150 and miR-638 were used. In an earlier study by our group, miR-638 had been shown to be a major contributor to melanoma growth [42], and miR-150 had been identified above. Indeed, PEI/antimiR-150 or PEI/antimiR-638 treatment led to a profound 40–50% reduction of tumor growth. PEI-complexed antimiR-638 was slightly more efficient than antimiR-150 (Fig. 5a, b). On the molecular level, the analysis of the explanted tumor tissues revealed alterations in the expression of murine double minute 4 (MDM4) which has been described previously as direct target of miR-150 [44]. The antimiR-mediated de-repression of MDM4 expression led to enhanced levels, as determined by quantitation of the total immunopositive area as well as by the mean MDM4 staining intensity (Fig. 5c).

### Anti-metastatic effect of PEI/antimiR treatment

**Fig. 6:**
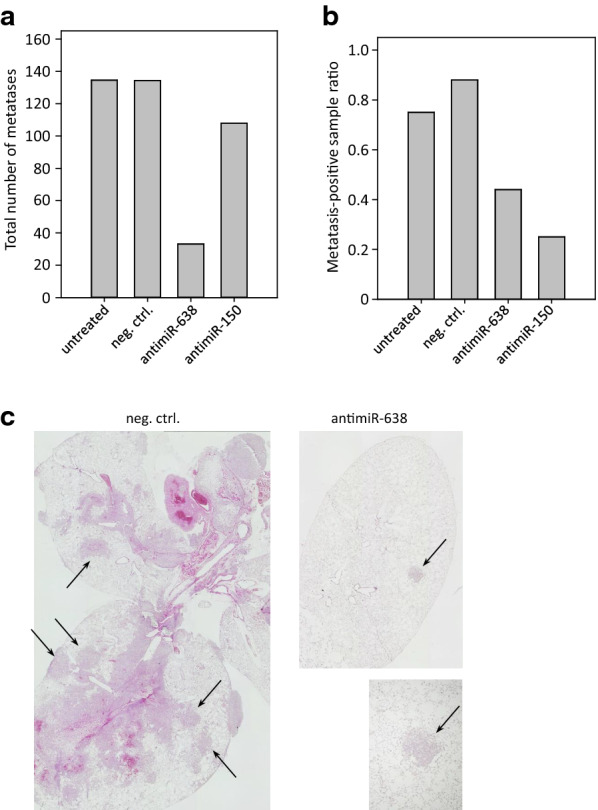
Treatment of distant melanoma metastases with antimiR/PEI complexes in mice. **a,b** For the analysis of anti-metastatic effects of PEI/antimiR-mediated inhibition, 5 × 10^6^ LOX melanoma cells were injected intravenously into the tail veins of NOD scid gamma mice. After 7 days, mice were randomized into specific treatment groups (antimiR-150, antimiR-638 and scrambled RNA). The PEI/antimiR complexes were injected intraperitoneally every 2 days. Lungs will be surgically removed after 4 weeks and lung metastasis was evaluated by counting of number of melanoma metastatic lesions in representative sections of each lung and the number of affected lungs. The total number of metastases per group and number of affected lung samples is given as bar graphs. **c** Representative examples of metastatic lesions in mouse lungs (arrows)

Since both, miR-638 and miR-150, have been implicated with metastasis ([42] and Fig. 4a, b), we also analyzed possible inhibitory effects of our PEI/antimiR complexes on the formation of metastases. For this, mice were i.v. injected with LOX cells prior to repeated treatment with PEI-complexed antimiR-638 or antimiR-150 vs. PEI-complexed negative control (scrambled RNA). Upon termination of the experiment after 4 weeks, lungs were surgically removed and paraffin sections were prepared. Analysis of serial sections by H&E staining revealed an inhibition in metastasis formation in the specific treatment groups, as indicated by lower total numbers of metastases. This was true with regard to the total number of metastases determined in all analyzed sections (Fig. 6a) as well as for the number of metastasis positive samples (Fig. 6b; given as metastasis-positive sample ratio). Metastatic lesions showed significant variation in diameters. Together these data showed that treatment of mice with PEI-complexed antimiRs showed a significant treatment response.

## Discussion

The development of non-viral RNA delivery tools as well as the selection of functionally relevant oncomiRs are pivotal requirements for miRNA inhibition strategies. In this paper, we establish PEI complexes as delivery platform for antimiR therapy. On purpose, we selected different in vivo models from different tumor entities to also demonstrate their broader applicability.

As therapeutic targets in prostate carcinoma (PCa), miR-141 and miR-375 were selected in this treatment study since both miRNAs have been found overexpressed in prostate cancer patients, also demonstrating their prognostic value [45–47]. In serum and plasma, a significant correlation with metastatic PCa was found [48]. High levels of miR-375 or miR-141 were associated with poor overall survival (OS), aggressive tumor characteristics and poor outcome [49, 50] in prostate cancer. The roles of both miRNAs in this tumor entity may be similar and interactive, based on common targets. Moreover, both miRNAs have been found to be associated with treatment outcome and may thus serve as predictors for castration resistant prostate cancer (CRPC) disease progression under docetaxel or abiraterone treatment [51]. Thus, despite contradictory findings in other tumor entities, pointing towards an ambivalent role of miR-141 in various human cancers [52–54], and one report on a dual role of miR-375 in prostate carcinogenesis [55], their inhibition could be expected to represent a valuable approach towards novel treatment regimens in prostate cancer, which has not been explored so far. Indeed, our in vitro studies support this notion, by demonstrating profound tumor cell growth inhibitory and pro-apoptotic effects of both antimiRs. Notably, this was also true for antimiR-375 in PC3 cells, despite an opposite previous finding on the role of miR-375 in this cell line [55]. Thus, our results held true in several (androgen-dependent or -independent) cell lines, with effects of miR-141 inhibition being somewhat stronger. While the combined inhibition of both miRNAs did not seem to produce additive or synergistic effects, thus being in line with the notion of their overlapping function, it is tempting to speculate that the combination of antimiR treatment with chemo- or androgen ablation therapy may in fact lead to further increases in treatment efficacies.

Likewise, two miRNAs were selected as targets in melanoma. MiR-150 has been established as potential biomarker with diagnostic power [56]. More specifically, it was found upregulated in plasma of melanoma patients compared with healthy controls [57], with a statistically significant postsurgical downregulation of miR-150 relative to pre-surgical samples [58]. However, conflicting results exist with regard to its oncogenic or tumor-suppressive function. On the one hand, miR-150 has been described to suppress proliferation, migration and invasion of melanoma cells through inhibition of SIX1 [59] and downregulation of MYB [60]. Concomitantly, a prognostic signature of six miRNAs including miR-150 has been identified, with high expression of these miRNAs associated with improved long-term survival of metastatic patients [61]. On the other hand, cell proliferation, migration, and invasion were found to be suppressed by miR-150 inhibitors, simultaneously promoting cell apoptosis and G2/M cell arrest via upregulation of PDCDC4 [62]. The latter findings regarding an oncogenic function of miR-150 are supported by our studies which demonstrate increased tumorigenesis upon miRNA transfection, while antimiR-150-mediated inhibition leads to decreased tumor growth. Notably, in our miRNA expression profiling data miR-150 also scored highest in the comparison LNM versus ME and DCM versus ME, thus emphasizing its potential role in tumor progression and metastasis. The observed tumor-inhibitory effects of antimiR-150 treatment in our setting is in line with its tumor promoting role. This might explain the failure to induce c-MYB upregulation which would have to be expected according to the above mentioned study, where miR-150 exerted a tumorsuppressive function [60]. The observed increase of MDM4 in locally induced melanoma tumors is in line with its previous description as a direct miR-150 target gene [44]. In addition, a positive activity exerted by MDM4 on stress-activated p53 levels and pro-apoptotic function has been described [63]. The complex MDM4/HIPK2/p53 can repress anti-apoptotic proteins that are frequently overexpressed and/or markers of tumor phenotype in human cancer [64].

The therapeutic inhibition of miRNAs involved in later tumor stages may be of particular therapeutic relevance. This is supported by the anti-metastatic effect of our PEI/antimiR-150 treatment, thus addressing a major goal in oncology: therapy or prevention of metastases. Additionally, miR-638 was selected which had been identified in our previous work as oncologically relevant by promoting melanoma metastasis and protecting melanoma cells from apoptosis and autophagy [42]. Indeed, inhibition of both miRNAs was found efficient in reducing primary melanoma growth as well as metastases.

The analysis of the tissue sections by immunostaining for direct target genes of the selected miRNAs revealed that the observed antimiR effects were indeed accompanied by target gene upregulation. Of course, it should be kept in mind that other genes will be affected by antimiR treatment as well, which may contribute to an even larger extent to the observed therapeutic effects. Thus, the underlying molecular consequences of antimiR treatment can be expected to be more complex. On the other hand, this broader specificity may also contribute to more prominent tumor-inhibitory effects, considering cancer as a pathway disease [65] dependent on the parallel action of different oncogenes. In this respect, antimiR therapy is comparable to miRNA replacement therapy, which has been introduced in preclinical studies [21].

Comparable to miRNA or siRNA therapy, issues related to the delivery of the full-length intact RNA molecules are still major obstacles in their therapeutic use. Various nanoparticle systems have been explored in this respect [66, 67]. While certain low molecular weight PEIs have been shown to provide sufficient efficacy in the absence of toxicity related to their higher molecular weight counterparts, they often show limitations with regard to small RNA molecules. In particular, siRNA molecules are often poorly complexed due to their short sequences and their double strand rigidity [68]. Our study provides evidence that PEI F25-LMW is sufficiently potent for antimiR complexation, mediating antimiR protection against degradation and excretion, delivery to the target tissue, cellular internalization and intracellular release. It thus provides the basis for therapeutic use of antimiRs.

Taken together, here we provide substantial evidence that nanoparticle complexed antimiRs may be used as specific treatment options in two different solid cancers entities. Future treatment options may include this treatment modality to enhance treatment response using classical chemotherapy or targeted treatment.

## Methods

### Materials

Branched low molecular weight (4–10 kDa) polyethylenimine PEI F25-LMW was prepared as described previously [18]. Cell Culture media, Dulbecco’s phosphate buffered saline (PBS) and trypsin were from Sigma Aldrich (Taufkirchen, Germany), and fetal calf serum (FCS) was purchased from Gibco Thermo Fisher (Darmstadt, Germany). Cell culture plastic and other disposable plastics were from Sarstedt (Nümbrecht, Germany). Chemically synthesized antimiRs (miRCURY LNA inhibitor; Exiqon/Qiagen, Hilden, Germany or 2′-OMe modified RNAs; Eurogentec, Seraing, Belgium) were used; see Additional file 3: Table S1 for additional information. Stable human prostate carcinoma cell lines PC3, DU145 and LNCaP as well as the human or mouse melanoma cell lines LOX, A375, SK-Mel-28, B16F10 and B16V were obtained from ATCC/LGC Promochem (Wesel, Germany) or the German Collection of Microorganisms and Cell Cultures (DSMZ), Braunschweig, Germany).

### Cell culture and cellular assays

All cell lines were cultured in a humid atmosphere at 37 °C and 5% CO_2_ in RPMI-1640 supplemented with 10% FCS*. *In vitro transfections were performed using Interferin (Polyplus, Illkirch, France) according to the manufacturer’s protocol for siRNA, with 10 or 20 nM antimiR. Cell proliferation was determined in 96-wells (2000 cells/well) using the colorimetric WST-1 assay (Roche) as described previously [69] or by cell counting using a hemocytometer (Neubauer chamber). For the assessment of colony formation capacity, cells were transfected 1 d prior to seeding at low density (1000 cells/well) in 6-well plates. To measure anchorage-independent proliferation, cells were seeded in 6-well plates and transfected as described above. 48 h after transfection, soft agar assays were performed as described previously [70]. Soft agars were run in triplicate wells and incubated under standard conditions. At the time points indicated, colonies > 50 µm were counted by at least two blinded investigators.

For the quantification of caspase-3/-7 activity, the bioluminescent Caspase-Glo^®^ 3/7 assay (Promega, Mannheim, Germany) was used. Cells were seeded in 96-well plates and transfected as described above. After 96 h cultivation under standard conditions, the Caspase-Glo^®^ assay was performed according to the manufacturer’s protocol. Luminescence was measured using a POLARstar Omega reader (BMG Labtec, Jena, Germany) after 1 h of incubation at room temperature in the dark. A WST-1 assay was performed in parallel on the same plate as described above, to normalize for variations in cell densities.

### Complex preparation

PEI/antimiR complexes were prepared using PEI F25-LMW as described previously for siRNA [24]. Briefly, for the preparation of complexes for a standard knockdown experiment in a 24 well format, 0.4 µg (80 pmol) antimiR per well were diluted in 12.5 µL HN buffer (10 mM HEPES, 150 mM NaCl in DEPC-H_2_O, pH 7.4). In a second tube, the appropriate PEI amount (3 µg for mass ratio 7.5, unless indicated otherwise) was dissolved in 12.5 µL HN buffer. The antimiR solution was then added to the polymer solution, briefly vortexed and incubated for 45 min at RT. Accordingly, 0.1 µg (20 pmol) antimiR were complexed in a final volume of 10 µL for the 96 well format. For in vivo experiments, larger complex amounts were prepared, aliquoted and stored at − 80 °C. Prior to use the complexes were thawed and incubated for 15 min.

### Photon Correlation Spectroscopy (PCS) and phase analysis light scattering (PALS)

Zeta potentials and particle sizes of complexes prepared as described above were measured as described previously [71]. Briefly, complexes containing 20 µg antimiR was diluted to 1.5 mL pure water prior to phase analysis light scattering (PALS) and photon correlation spectroscopy (PCS), using a Brookhaven ZetaPALS system (Brookhaven Instruments, Holtsville, USA). The data were analyzed using the manufacturer’s software and applying a viscosity and refractive index of pure water at 25 °C. Zeta potentials were measured in ten runs, with each run containing ten cycles, and applying the Smoluchowski model. For size determination, the complexes were analyzed in five runs with a run duration of 1 min. Results are expressed as intensity weighted mean diameter from different experiments. Additionally, sizes were analyzed by nanoparticle tracking analysis (NTA) using NanoSight LM10 (Malvern) equipped with a 640 nm sCMOS camera, software NTA 3.0, and an antimiR concentration of 1 µg/mL.

### Electron microscopy

For transmission electron microscopy (TEM), 300-mesh nickel grids were coated with a thin film made of 1% pioloform solution (Plano, Wetzlar, Germany). For increasing hydrophilicity, UV radiation for 30 min and pre-incubation in a dH_2_O droplet for 5 min were performed. Grids were incubated for 10 min in 10 µL suspension containing 0.5 µg antimiR complexed with PEI F25-LMW three times and air-dried overnight. The samples were negatively stained with 1% aqueous uranyl acetate. After rinsing and extensive drying, transmission electron microscopy (TEM Libra 120, Zeiss, Oberkochen, Germany) was done in bright field mode at 80 keV, and images were taken with a 4-megapixel CCD camera equipped with a YAG scintillator and ISP software (TRS Tröndle, Moorenweis, Germany).

### Complexation efficacy and complex stability

For the determination of complexation efficacy by agarose gel electrophoresis, 0.5 µg antimiR was complexed with PEI at the different weight ratios indicated in Fig. 2a in 40 µL HN buffer or in 5% glucose, 10 mM HEPES, pH 7.4. The complexes were mixed with 10× loading dye and separated onto a 2% agarose gel pre-stained with 1× Sybr™ Gold in TAE buffer running buffer. Complex stabilities were measured by heparin displacement assay. PEI/antimiR complexes at a ratio of 7.5:1 were prepared as described above and mixed with increasing amounts of heparin as indicated in Fig. 2b. After an incubation of 30 min, the samples were analyzed by agarose gel electrophoresis as above.

The stabilities of uncomplexed antimiR and PEI/antimiR complexes were assessed by incubation with RNase A, serum and under acidic conditions. For this, 0.5 µg uncomplexed or complexed antimiR was incubated for 4 h at 37 °C with 25% (v/v) FCS, 0.5 µg RNase A or the combination of both, as indicated in the figure. To mimic conditions in the lysosome, incubations were also performed in 50% (v/v) artificial lysosomal fluid (ALF) [72] for 2 h at 37 °C. Since antimiRs are chemically modified with LNA nucleotides, an unmodified siRNA was run in parallel, for direct comparison and as positive control. For complex decomposition, 40 units RNase inhibitor and 100 units heparin were added. AntimiR or siRNA integrity was analyzed by agarose gel electrophoresis as described above.

### Complex uptake and intracellular localization

The uptake of PEI complexes containing Cy3-labeled antimiR or FAM-labeled antimiR (Additional file 3: Table S1) was analyzed by flow cytometry and confocal microscopy. For flow cytometry, PC3 cells were seeded at a density of 35,000 cells per 24 well and transfected the next day with PEI complexes containing 0.4 µg antimiR-Cy3. After 48 h, the cells were harvested and washed two times using FACS buffer (PBS, 2% FCS) and centrifugation at 3000 rpm for 3 min. Next, the cells were resuspended in 600 µL FACS buffer and analyzed in an Attune Acoustic Focusing Cytometer (Applied Biosystems, Foster City, CA). 20,000 events were measured in the vital gate using the Attune software (V2.1.0).

Optical evaluation of PEI complex uptake was performed by confocal laser scanning microscopy, using a Leica SP8 and the Leica Application Suite software (Wetzlar, Germany). For this, PC3 cells were seeded in a CELLview 4 compartment cell culture dish (Greiner Bio-One, Frickenhausen, Germany) at a density of 20,000 cells. The next day, the cells were transfected with PEI complexes containing 0.1 µg antimiR-Cy3 and analyzed after 48 h. For co-localization studies, the lysosomes were stained with LysoTracker Green DND-26 (Thermo Fisher) at a final concentration of 50 nM according to the manufacturer’s protocol. Colocalization was analyzed using the Fiji software and the colocalization plugin, for calculating the Pearson correlation coefficient from four images (20× magnification).

### MicroRNA expression profiling

TaqMan low-density arrays (TLDA; human microRNA Cards A v2.1 & B v2.0, Applied Biosystems, Darmstadt, Germany) were used for measuring the expression of 667 human miRNAs from miRBase version v.10 with an Applied Biosystems 7900HT. Raw data were exported using SDS Relative Quantification Software version 2.2.2 (Applied Biosystems) with automatic baseline and threshold settings.

### Immunofluorescence

6 μm cryosections of locally induced melanoma tumors in mice were stained with primary antibodies directed against MDM4 (anti-MDM4 antibody produced in rabbit, HPA018919-100 UL, Sigma Aldrich, Munich, Germany), diluted in PBS, 0.1% Tween 20, 3% bovine serum albumin, and appropriate secondary antibodies (IRDye^®^ 680RD Goat anti-Rabbit IgG (#926-68071). Washing steps were performed with PBS, 0.1% Tween-20. Cell nuclei were counterstained with DAPI (Merck, Darmstadt, Germany). Immunofluorescence images were taken with a BZ-9000Z microscope camera (Keyence, Neu-Isenburg, Germany). The number of labeled cells was calculated with the corresponding Keyence analyzer software version 2.2 (Keyence).

### Western blot analysis

Lysates containing 15 µg total protein were separated by SDS-PAGE (8%) and transferred onto a nitrocellulose membrane (GE Healthcare, Freiburg, Germany) by electroblotting. Primary antibodies used were monoclonal rat anti-Sec23A (clone 2H4, provided by E. Kremmer, Munich, Germany; 1:15) polyclonal rabbit anti-PHLPP1 (A304-029A; Bethyl laboratories, Montgomery, TX, USA; 1:1000) and monoclonal rabbit anti-GAPDH (clone 14C10, NEB Cell Signaling, Frankfurt, Germany; 1:1000). Secondary anti-rat or anti-rabbit antibodies conjugated with horseradish peroxidase were purchased from Jackson ImmunoResearch (Suffolk, UK) and used at a concentration of 1:5000. Protein bands were revealed using enhanced chemiluminescence in a LAS-4000 chemiluminescence detection system (GE Healthcare, Munich, Germany). Densitometry of Western blot pictures was performed using ImageJ software, and the expression of Sec23A and PHLPP1 protein was normalized to GAPDH as the housekeeping control protein.

### In vitro colony formation assay

Candidate miRNAs found upregulated in the TaqMan^®^ low-density arrays of melanoma samples were ectopically overexpressed in three different melanoma cells lines, SK-Mel-28 (human), A375 (human) and B16V (mouse), by Lipofectamine (ThermoFisher Scientific)-mediated transfection of miRNA mimics. The miRNA mimics (miRIDIAN microRNA hsa-miR-638 shMimic (#300029051) or mirVana^®^ miRNA mimic hsa-miR-150-5p (#MC10070) were purchased from ThermoFisher Scientific. Melanoma cells were seeded at a low density in six-well plates and allowed to grow for 20 days. The numbers of colonies were counted, and differences between miR-150 and control RNA were analyzed using Student’s t-test.

### In vivo tumor growth of pre-transfected melanoma cells

All animal studies were performed according to the national regulations and approved by the local authorities (Landesdirektion Sachsen). B16V mouse melanoma cells were transfected with the miR-150 mimic. Control cells were transfected with scrambled RNA controls (ThermoScientific). A total of 5 × 10^5^ cells suspended in 50 μL of RPMI were injected subcutaneously at the back of C57BL6N mice (n = 10 mice/miRNA). For this, the mice were anaesthetized by injecting 100 μL of ketamine prior to tumor implantation. Mice were sacrificed, and after 14 days volumes of surgically removed tumors were measured and tumors weighted.

To further test the influence of candidate miRNAs on in vivo melanoma growth in mice, B16V melanoma cells were transfected with antimiRs against miR-150 or antimiR-control oligos (mirVana™ miRNA Inhibitors; ThermoFisher Scientific), prior to cell injection as above. Mice were sacrificed after 14 days and surgically removed tumors were weighted.

### In vivo tumor therapy of locally induced tumors

Athymic nude mice (Foxn1nu, Charles River Laboratories, Sulzfeld, Germany) were kept at 23 °C in a humidified atmosphere, 12 h light/dark cycle, with standard rodent chow and water ad libitum. For the generation of tumor xenografts, 5 × 10^6^ PC3 cells or 5 × 10^6^ LOX cells in 150 µL PBS were injected subcutaneously (s.c.) into both flanks of mice. When tumors reached sizes of ~ 85–90 mm^3^, mice were randomized into specific treatment and control treatment groups (PC3 xenografts, n = 10 tumors/group; LOX xenografts, 14–20 tumors/group). The PEI/antimiR complexes were prepared as described above and complexes containing 10 µg antimiR were injected i.p. every 2–3 days at the time points indicated in the figure. Tumor sizes were determined by measuring all three dimensions. Upon termination of the in vivo studies, mice were euthanized and tumor tissues were collected for analysis of protein levels of miRNA target genes.

### In vivo metastasis model and therapy

For analysis of anti-metastatic effects of the PEI/antimiR-mediated inhibition, 5 × 10^6^ LOX melanoma cells were injected intravenously into the tail veins of NOD scid gamma mice. After 7 days, mice were randomized into specific treatment groups (antimiR-150, antimiR-638, or scrambled RNA) of 10 mice per group, and treatment with PEI-complexed antimiRs was performed. For this, the PEI/antimiR complexes were prepared as described above and injected intraperitoneally every 2 days. Lungs were surgically removed after 4 weeks and lung metastasis was evaluated by counting of number of melanoma metastatic lesions in representative sections of each lung and the number of affected lungs. Sections were evaluated by light microscopy.

### Statistics

For statistic evaluation a Student’s t-test was used. Values of specific treatments are always compared to negative control treatment (neg.ctrl.), with *p < 0.05, **p < 0.01 and ***p < 0.001. Values are given as means ± standard error of the mean (SEM).

## Supplementary information

**Additional file 1: Fig. S1.** **a** Uptake of FAM-labeled, complexed (red) or uncomplexed antimiRs (blue) in PC3 cells, as determined by flow cytometry. Histograms and scatter plots show the direct comparison (upper panels). The lower panel reveals the absence of any uptake of uncomplexed antimiR, since results are indistinguishable from untreated cells (black, lower left). **b** Determination of cell viability upon treatment of PC3 cells with complexed (left) vs. uncomplexed antimiRs (right).

**Additional file 2: Fig. S2.** **a** Determination of complex stability and antimiR integrity in the presence of FCS and/or RNase. Bands represent the antimiR after the indicated treatments, either uncomplexed (lanes 1–4), complexed (lanes 9–10) or complexed and subsequently released by heparin displacement (lanes 5–8). **b** The same experiment as in a, with siRNA instead of antimiR for direct comparison. **c** Complex stability and antimiR integrity in the presence of artificial lysosomal fluid (ALF; experimental procedure as above).

**Additional file 3: Table S1.** Details on antimiRs used in this study.

## Data Availability

All data generated or analysed during this study are included in this published article and/or are available from the corresponding author on reasonable request.

## Abbreviations

AntimiR: Anti-miRNA oligonucleotide
DCM: Distant cutaneous metastases
LNM: Lymph node metastases
ME: Primary melanocytes
PALS: Phase analysis light scattering
PCa: Prostate carcinoma
PCS: Photon Correlation Spectroscopy
PEI: Polyethyleneimine
PM: Primary malignant melanomas
