# Supplementary material for: Nanoparticle-complexed antimiRs for inhibiting tumor growth and metastasis in prostate carcinoma and melanoma
Source: J Nanobiotechnology. 2020 Nov 23;18:173. doi: 10.1186/s12951-020-00728-w (PMC7685669; doi:10.1186/s12951-020-00728-w)
Supplement: Supplementary file 1 — Additional file 1: Fig. S1. a Uptake of FAM-labeled, complexed (red) or uncomplexed antimiRs (blue) in PC3 cells, as determined by flow cytometry. Histograms and scatter plots show the direct comparison (upper panels). The lower panel reveals the absence of any uptake of uncomplexed antimiR, since results are indistinguishable from untreated cells (black, lower left). b Determination of cell viability upon treatment of PC3 cells with complexed (left) vs. uncomplexed antimiRs (right). [file 12951_2020_728_MOESM1_ESM.pptx]

## Slide 1
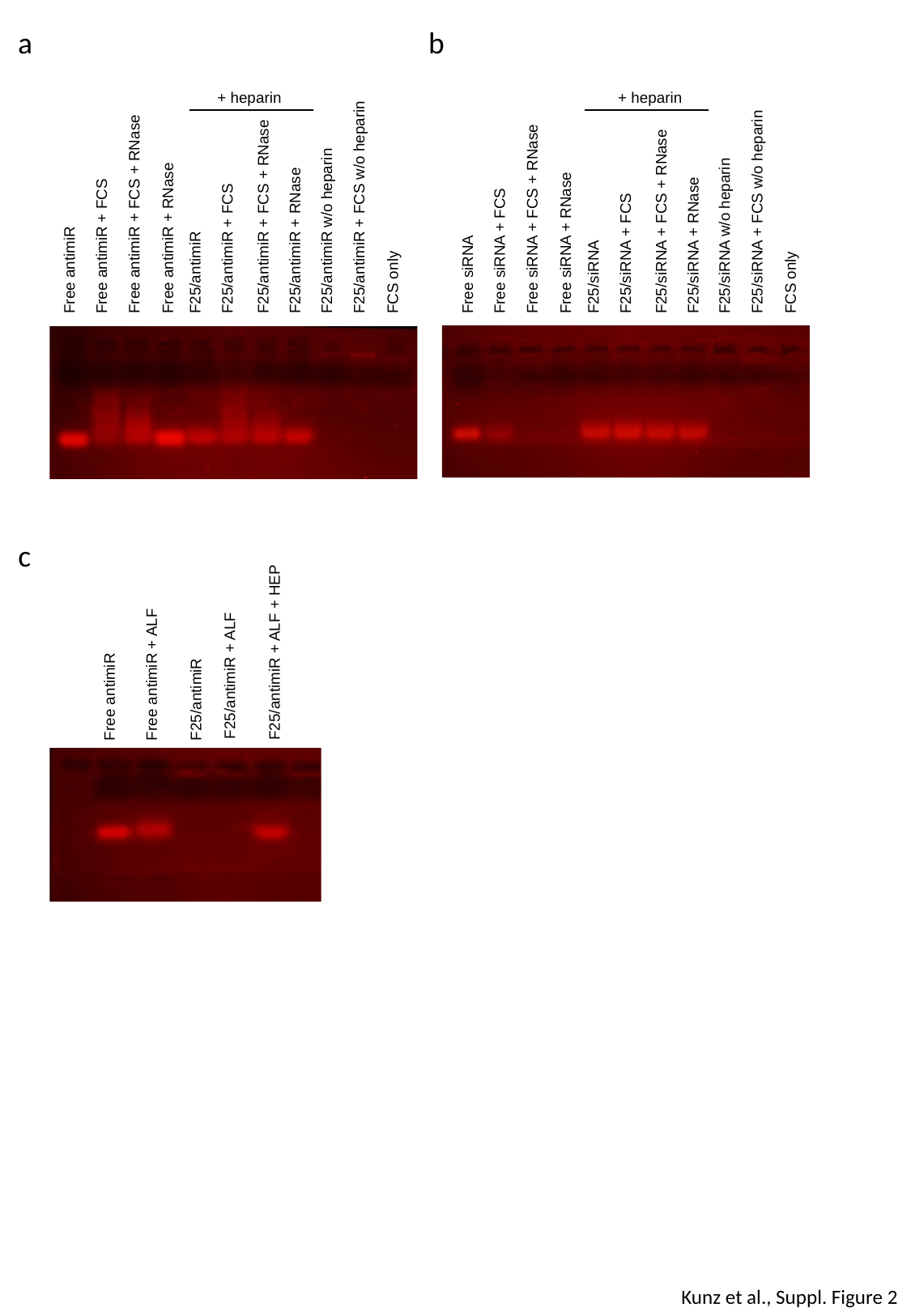

a
b
+ heparin
+ heparin
Free antimiR + FCS + RNase
Free antimiR + RNase
F25/antimiR
F25/antimiR + FCS
F25/antimiR + FCS + RNase
F25/antimiR + RNase
F25/antimiR w/o heparin
F25/antimiR + FCS w/o heparin
FCS only
Free siRNA + FCS + RNase
Free siRNA + RNase
F25/siRNA
F25/siRNA + FCS
F25/siRNA + FCS + RNase
F25/siRNA + RNase
F25/siRNA w/o heparin
F25/siRNA + FCS w/o heparin
FCS only
Free antimiR + FCS
Free siRNA + FCS
Free antimiR
Free siRNA
c
F25/antimiR + ALF
F25/antimiR + ALF + HEP
F25/antimiR
Free antimiR + ALF
Free antimiR
Kunz et al., Suppl. Figure 2
