# Supplementary material for: Nanoparticle-complexed antimiRs for inhibiting tumor growth and metastasis in prostate carcinoma and melanoma
Source: J Nanobiotechnology. 2020 Nov 23;18:173. doi: 10.1186/s12951-020-00728-w (PMC7685669; doi:10.1186/s12951-020-00728-w)
Supplement: Supplementary file 2 — Additional file 2: Fig. S2. a Determination of complex stability and antimiR integrity in the presence of FCS and/or RNase. Bands represent the antimiR after the indicated treatments, either uncomplexed (lanes 1–4), complexed (lanes 9–10) or complexed and subsequently released by heparin displacement (lanes 5–8). b The same experiment as in a, with siRNA instead of antimiR for direct comparison. c Complex stability and antimiR integrity in the presence of artificial lysosomal fluid (ALF; experimental procedure as above). [file 12951_2020_728_MOESM2_ESM.docx]

Kunz et al., Suppl. Table 1: Details on antimiRs used in this study

| **antimiR** | **modification** | **sequence** | **purification** | **vendor** |
| --- | --- | --- | --- | --- |
|  |  |  |  |  |
| antimiR-638 | * 2'OMe RNA | C*C*C*-UAG-GCC-GCC-ACC-CGC-CCG-CGA-UCC-CUG*-C*C*G | HPLC-RP | Eurogentec  Seraing, Belgium |
| antimiR-150 | * 2'OMe RNA | C*C*A*-GCA-CUG-GUA-CAA-GGG-UUG-GGA-GAC*-A*G*G | HPLC-RP | Eurogentec  Seraing, Belgium |
| neg. ctrl. antimiR | * 2'OMe RNA | C*U*C*-GCC-CAC-CGU-CGC-UAC-CCC-CCA-CCG-GGC*-C*G*G | HPLC-RP | Eurogentec  Seraing, Belgium |
| antimiR-141 | * Phosphothioate backbone, LNA enhancement | T*T*T*A*C*C*A*G*A*C*A*G*T*G*T*T | HPLC | Exiqon Vedbaek, Denmark |
| antimiR-375 | * Phosphothioate backbone, LNA enhancement | G*A*G*C*C*G*A*A*C*G*A*A*C*A*A | HPLC | Exiqon Vedbaek, Denmark |
| neg. ctrl. antimiR | * Phosphothioate backbone, LNA enhancement | A*C*G*T*C*T*A*T*A*C*G*C*C*C*A | HPLC | Exiqon Vedbaek, Denmark |
| Cy3-neg.ctrl.  antimiR | * Phosphothioate backbone, LNA enhancement | Cy3*TAACACGTCTATACGCCCA | HPLC | Exiqon Vedbaek, Denmark |
| FAM-neg.ctrl.  antimiR | * Phosphothioate backbone, LNA enhancement | FAM*TAACACGTCTATACGCCCA | HPLC | Qiagen, Hilden, Germany |
