# Supplementary material for: Nanoparticle-complexed antimiRs for inhibiting tumor growth and metastasis in prostate carcinoma and melanoma
Source: J Nanobiotechnology. 2020 Nov 23;18:173. doi: 10.1186/s12951-020-00728-w (PMC7685669; doi:10.1186/s12951-020-00728-w)
Supplement: Supplementary file 3 — Additional file 3: Table S1. Details on antimiRs used in this study. [file 12951_2020_728_MOESM3_ESM.pptx]

## Slide 1
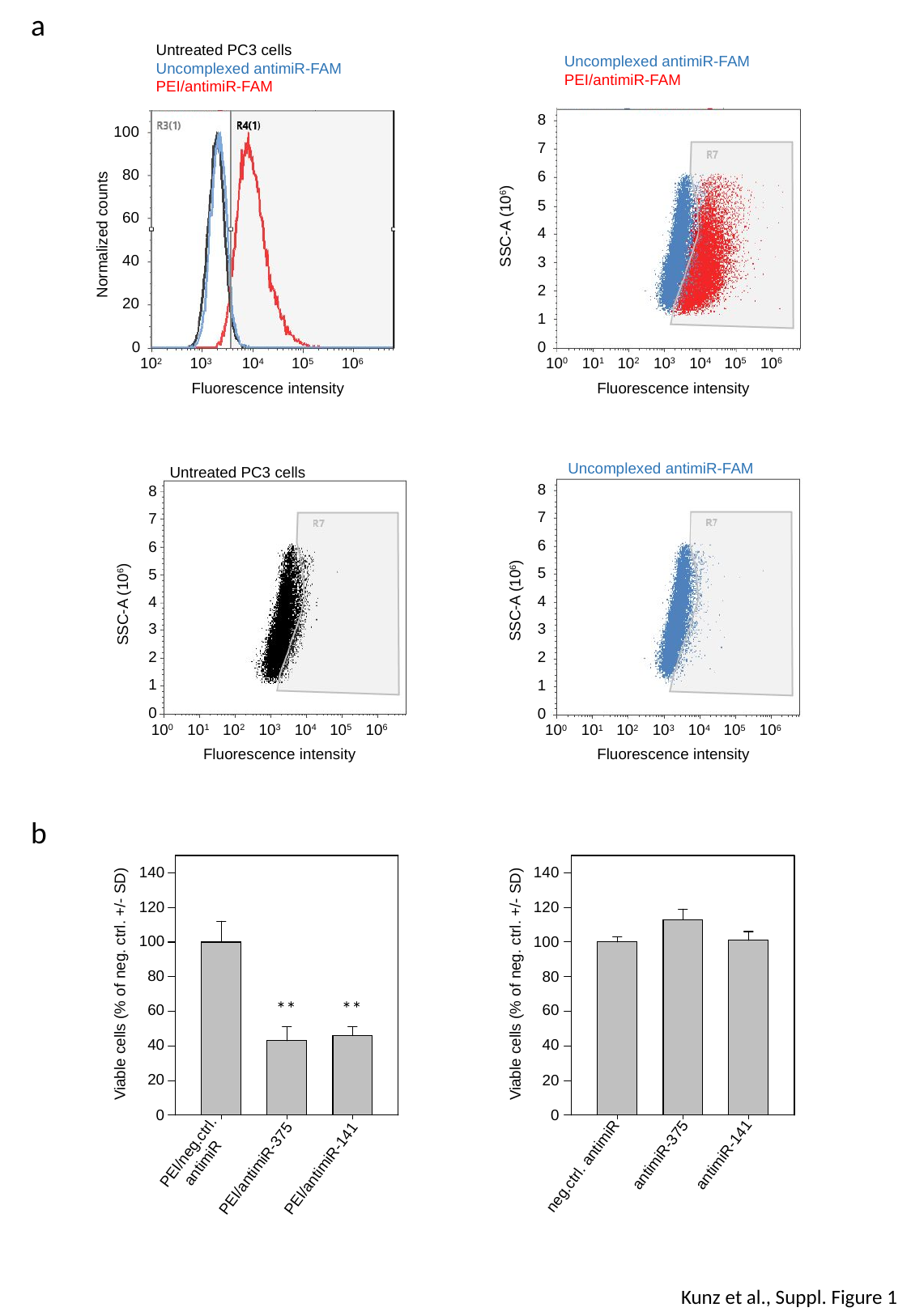

a
Untreated PC3 cells
Uncomplexed antimiR-FAM
PEI/antimiR-FAM
Uncomplexed antimiR-FAM
PEI/antimiR-FAM
8
100
7
80
6
5
60
SSC-A (106)
4
Normalized counts
40
3
2
20
1
0
0
100
101
102
103
104
105
106
102
103
104
105
106
Fluorescence intensity
Fluorescence intensity
Uncomplexed antimiR-FAM
Untreated PC3 cells
8
8
7
7
6
6
5
5
SSC-A (106)
4
4
SSC-A (106)
3
3
2
2
1
1
0
0
100
101
102
103
104
105
106
100
101
102
103
104
105
106
Fluorescence intensity
Fluorescence intensity
b
140
140
120
120
100
100
80
80
Viable cells (% of neg. ctrl. +/- SD)
Viable cells (% of neg. ctrl. +/- SD)
**
**
60
60
40
40
20
20
0
0
PEI/neg.ctrl. antimiR
antimiR-375
antimiR-141
PEI/antimiR-375
PEI/antimiR-141
neg.ctrl. antimiR
Kunz et al., Suppl. Figure 1
